# Developmental remodeling of relay cells in the dorsal lateral geniculate nucleus in the absence of retinal input

**DOI:** 10.1186/s13064-015-0046-6

**Published:** 2015-07-15

**Authors:** Rana N. El-Danaf, Thomas E. Krahe, Emily K. Dilger, Martha E. Bickford, Michael A. Fox, William Guido

**Affiliations:** Departments of Neuroscience, Neurobiology Section in the Division of Biological Sciences, University of California, San Diego, La Jolla, CA 92093 USA; Department of Anatomy and Neurobiology, Virginia Commonwealth University Medical Center, Richmond, VA 23298 USA; Society for Neuroscience, Washington D.C., 20005 USA; Department of Anatomical Sciences and Neurobiology, University of Louisville School of Medicine, Louisville, KY 40292 USA; Virginia Tech Carilion Research Institute, Roanoke, VA 24016 USA; Department of Biological Sciences, Virginia Tech, Blacksburg, VA 24061 USA

**Keywords:** Dorsal lateral geniculate nucleus, Retinogeniculate pathway, Relay cells, Retinal ganglion cells, Dendritic development, *math5 null*

## Abstract

**Background:**

The dorsal lateral geniculate nucleus (dLGN) of the mouse has been an important experimental model for understanding thalamic circuit development. The developmental remodeling of retinal projections has been the primary focus, however much less is known about the maturation of their synaptic targets, the relay cells of the dLGN. Here we examined the growth and maturation of relay cells during the first few weeks of life and addressed whether early retinal innervation affects their development. To accomplish this we utilized the *math5* null (*math5*^*−/−*^) mouse, a mutant lacking retinal ganglion cells and central projections.

**Results:**

The absence of retinogeniculate axon innervation led to an overall shrinkage of dLGN and disrupted the pattern of dendritic growth among developing relay cells. 3-D reconstructions of biocytin filled neurons from *math5*^*−/−*^ mice showed that in the absence of retinal input relay cells undergo a period of exuberant dendritic growth and branching, followed by branch elimination and an overall attenuation in dendritic field size. However, *math5*^*−/−*^ relay cells retained a sufficient degree of complexity and class specificity, as well as their basic membrane properties and spike firing characteristics.

**Conclusions:**

Retinal innervation plays an important trophic role in dLGN development. Additional support perhaps arising from non-retinal innervation and signaling is likely to contribute to the stabilization of their dendritic form and function.

## Background

The dorsal lateral geniculate nucleus (dLGN) of the mouse thalamus has become a powerful model system to understand visual circuit development [23, 26, 27]. It has been especially useful for delineating the mechanisms underlying the establishment of the retinogeniculate pathway. A crucial element of this pathway is the synaptic target of retinal ganglion cells (RGCs), the relay cells of dLGN. These neurons serve as the principal conduit of information between the retina and visual cortex. Additionally, dLGN relay cells are the major site of convergence for a number of non-retinal inputs that work in concert to modulate the gain of retinogeniculate transmission in a state dependent manner [5, 48, 49].

Despite playing such a key role in visual processing, until recently little was known about the structural and functional composition of mouse dLGN relay cells. We found that mouse dLGN relay cells have highly stereotypic dendritic architecture and are readily classified as having X -, Y- or W-like profiles [30]. The distinguishing features of their dendritic morphology develop remarkably early in postnatal life. After the first postnatal week relay neurons have highly complex dendritic fields that already begin to resemble their adult counterparts. Accompanying this growth is the rapid maturation of their active membrane properties and spike firing characteristics. Such coordination enables relay cells to receive, integrate, and transmit retinal signals accurately by the time of natural eye opening [19, 28, 30, 36], when retinal activity switches from spontaneous to visually evoked [18, 53].

What remains unexplored is an understanding of the mechanisms that contribute to the development of relay cells. A prevailing view relates to the “synaptotrophic” hypothesis, which underscores the necessity of early synapse formation as a driving force for neuronal maturation (reviewed in [14, 56]). A likely candidate for dLGN relay cells is the support provided by retinal input [15]. These axons innervate the dLGN at perinatal ages, a time just after the nucleus takes shape and neuronal differentiation is completed [1, 22, 28]. Soon after birth newly formed axon terminals form functional synapses with dLGN cells [28, 40], and by postnatal week 2 retinogeniculate synapses begin to take on adult-like profiles [5].

A number of studies have adopted a loss of function approach to assess whether early retinal input and synapse formation contribute to dLGN development. However many of the manipulations to remove or silence retinal input did not focus on the development of relay cells per se [6, 25, 62], or more importantly, were done well after the time of early retinal innervation and synapse formation [6, 44, 47, 52]. Past attempts to employ a genetic form of deafferentation have also been problematic since “eyeless” phenotypes often involve a polygenic form of inheritance and are accompanied by other mutations that may have an indirect impact on neuronal development [16, 55, 58].

To overcome these issues we employed a relatively novel genetic form of retinal deafferentation by taking advantage of the *math5* null mutant mouse (*math5*^*−/−*^*).* Math5 is a basic helix-loop-helix (bHLH) gene that is expressed in the retina starting at embryonic day (E) 11 and is essential for the differentiation of retinal progenitor cells into RGCs [8]. As a consequence, *math5*^*−/−*^ exhibits a wholesale loss (>95 %) of RGCs [9, 41, 60], as well as a failure of the surviving cells to form an optic nerve [9, 10, 61]. Thus, this form of genetic deafferentation ensures that dLGN is devoid of retinal innervation even prior to perinatal times when retinal axons normally enter the nucleus. Here we made use of this mouse along with age matched wild types (WT) to understand whether retinal innervation affects the development of dLGN relay cells.

## Results

### Math5 expression in WT retina and dLGN

**Fig. 1:**
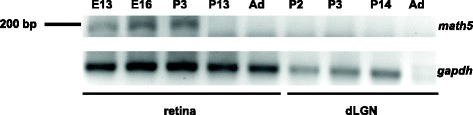
*Math5* expression in retina and dLGN of WT mouse. RT-PCR showing the expression of *math5* in WT retina and dLGN at different embryonic (E) and postnatal (P) ages. *Math5* expression is transient and restricted to the retina, appearing between E13 and P3. Note the absence of *math5* expression in dLGN at all ages. *Gapdh* (glyceraldehyde-3-phosphate dehydrogenase) is used as a control

*Math5* mRNA encodes a transcription factor that specifies RGC fate [8, 9, 60]. Embryonically, *math5* is expressed in the retina as well as the tenth cranial ganglion [8]. In the retina, *math5* is developmentally regulated, first appearing at E11, continuing through birth but absent in the adult [8, 9, 60]. However, there are some reports of *math5* expression in adult brain regions such as cerebellum and the ventral cochlear nucleus [45]. A closer examination of *math5* expression in central visual targets such as dLGN is lacking. Here we examined *math5* expression in the developing retina and dLGN using RT-PCR (Fig. 1; retina: *n* = 2 at each age; dLGN: *n* = 10 per age). As expected, in WT mice, *math5* was expressed in the retina between E13-P3, but absent at P13 and in the adult. Moreover, in WT dLGN we found no evidence of *math5* expression at any of the ages tested (e.g., P2, 3, 14, adult). Thus any reported changes observed among developing relay cells in *math5*^*−/−*^ cannot be attributed to the lack of *math5* in dLGN neurons, but rather is due to a direct consequence of RGC elimination.

### Absence of retinal input in math5^−/−^

**Fig. 2:**
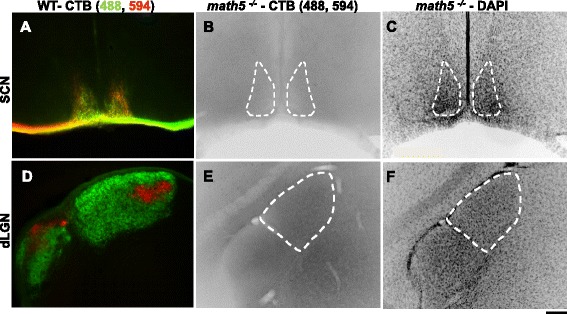
Absence of retinal projections in SCN and dLGN of *math5* ^*−/−*^. Anterograde labeling of retinal projections with CTB in the SCN and dLGN of WT (**a**, **d**) and *math5* ^*−/−*^ (**b**, **e**) adult mice. Retinal projections are visualized by injecting CTB conjugated to Alexa 488 (green; contralateral projections) in one eye and Alexa 594 (red; ipsilateral projections) in the other eye. Shown are coronal sections depicting SCN and dLGN for WT (**a**, **d**) and *math5* ^*−/−*^ (**b**, **e**). Inverted grey scale images in B and E depict the complete absence of CTB labeling in *math5* ^*−/−*^. (**c**, **f**) Corresponding DAPI images for sections (**b**) and (**e**). Dashed lines outline the boundaries of dLGN and SCN, and scale bar = 200 μm

While *math5*^*−/−*^ mice appear to lack an optic nerve, it is not clear whether the few remaining RCGs grow axons that enter the brain and innervate retino-recipient targets ([9, 10, 60, 61], but see [54]). To test for this possibility, the anterograde tracer CTB conjugated to different Alexa fluorescent dyes was injected into each eye of *math5*^*−/−*^ and WT mice (Fig. 2). This technique allows for the visualization of retinal terminal fields in central visual structures [28]. In WT mice, robust labeling of retinal terminals was apparent in all retino-recipient targets. For example in Fig. 2a, retinal axons from each eye innervated the suprachiasmatic nucleus (SCN) and formed overlapping terminal fields, whereas in dLGN they formed non-overlapping eye specific domains (Fig. 2d). By contrast, eye injections of CTB made in *math5*^*−/−*^ between P2-P48 (*n* = 8) failed to reveal any labeled elements in regions that correspond to optic nerve, optic tract or retino-recipient targets such as SCN or dLGN (Fig. 2b, e; see also [10, 61]).

**Fig. 3:**
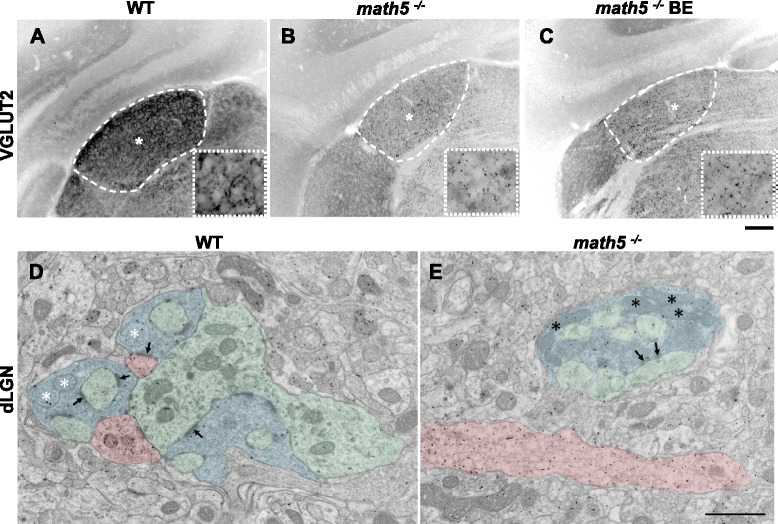
Absence of retinal terminals in dLGN of *math5* ^*−/−*^. **a**-**c** Coronal sections of dLGN showing the labeling pattern of the retinal terminal marker vesicular glutamate transporter 2 (VGluT2) in WT (**a**), *math5* ^*−/−*^ (**b**), and *math5* ^*−/−*^ seven days after binocular enucleation (**c**). At P14, there is strong expression of VGluT2 in WT and almost a complete absence in *math5* ^*−/−*^ (**b**), comparable to levels observed after binocular enucleation (**c**). Insets show high power images corresponding to the area denoted by the asterisk. Dashed lines in (**a**), (**b**), and (**c**) outline the border of dLGN, and scale bar for (**a**, **b**, **c**) = 200 μm, and 110 μm for the insets. **d-e**) Electron microscopy images revealing the ultrastructure of the dLGN in WT (**d**) and *math5* ^*−/−*^ mice (**e**). **d** Retinal terminals in WT mice (blue) include distinctive pale mitochondria (white asterisks). These terminals primarily synapse (arrows) on non-GABAergic dendrites (green), which often extend small protrusions into the presynaptic retinal terminals. E) In *math5* ^*−/−*^ mice, the dLGN contains no terminals with pale mitochondria. Instead, large profiles (blue) with dark mitochondria (black asterisks) form synaptic arrangements that are similar to retinal terminals, including contacts on non-GABAergic dendritic protrusions (green). GABAergic profiles (pink) are identified by a high density of overlying gold particles. Scale bar = 1 μm and applies to both panels

To further confirm the absence of retinal innervation in the dLGN, we used immunohistochemistry to detect the type 2 vesicular glutamate transporter (VGluT2), a reliable marker for retinal terminals in dLGN [21, 24, 31] (Fig. 3a). In a P14 *math5*^*−/−*^ mouse, there was almost a complete absence of VGluT2 in dLGN (Fig. 3b). The weak and sparse labeling we did detect was similar to the labeling pattern seen after a 7-day binocular enucleation (Fig. 3c), suggesting that the trace amounts of VGluT2 in *math5*^*−/−*^ dLGN, were of non-retinal origin [21, 24].

An ultrastructural analysis of the types of synapses found in dLGN of *math5*^*−/−*^ mice confirmed these findings (Fig. 3d-e). To distinguish excitatory from inhibitory profiles, we labeled those that contained gamma-aminobutyric acid (GABA) using an antibody that was subsequently tagged with gold particles. In WT mice, retinogeniculate terminals are characterized as large non-GABAergic profiles that contain round vesicles and pale mitochondria (RLP profiles, Fig. 3d, blue) [5]. In a sample of images from the dLGN of a WT mouse, (20 images at P21), we identified 29 RLP profiles with a mean area of 0.95 ± 0.11 μm^2^. Other non-GABAergic profiles were also abundant (*n* = 49) and had an average area of 0.51 ± 0.4 μm^2^; see also [5]. By contrast, in a sample of images from an age-matched *math5*^*−/−*^ (20 images), we failed to detect any RLP profiles. However, the overall population of non-GABAergic terminals present in *math5*^*−/−*^ mice (*n* = 46) was comparable in size to WT (WT, 0.68 ± 0.05 μm^2^ vs. *math5*^*−/−*^, 0.67 ± 0.08 μm^2^, Student’s *t*-test, *p* = 0.97). Interestingly, in *math5*^*−/−*^ mice, we noted the presence of non-GABAergic terminals characterized by having round vesicles, large profiles and dark mitochondria (RLD profiles) (Fig. 3e, blue, [20]). These so-called RLD profiles, which appear to supplant RLP profiles in *math5*^*−/−*^ mice have been observed in enucleated, anophthalmic and microphthamic strains of mice, but their origin is yet to be determined [16, 29, 63]. Taken together, these results indicate that the dLGN of *math5*^*−/−*^ mice serves as a suitable model for studying the development of relay cells in the absence of retinal innervation and signaling.

### Cytoarchitecture of the developing dLGN in WT and math5^−/−^ mice

**Fig. 4:**
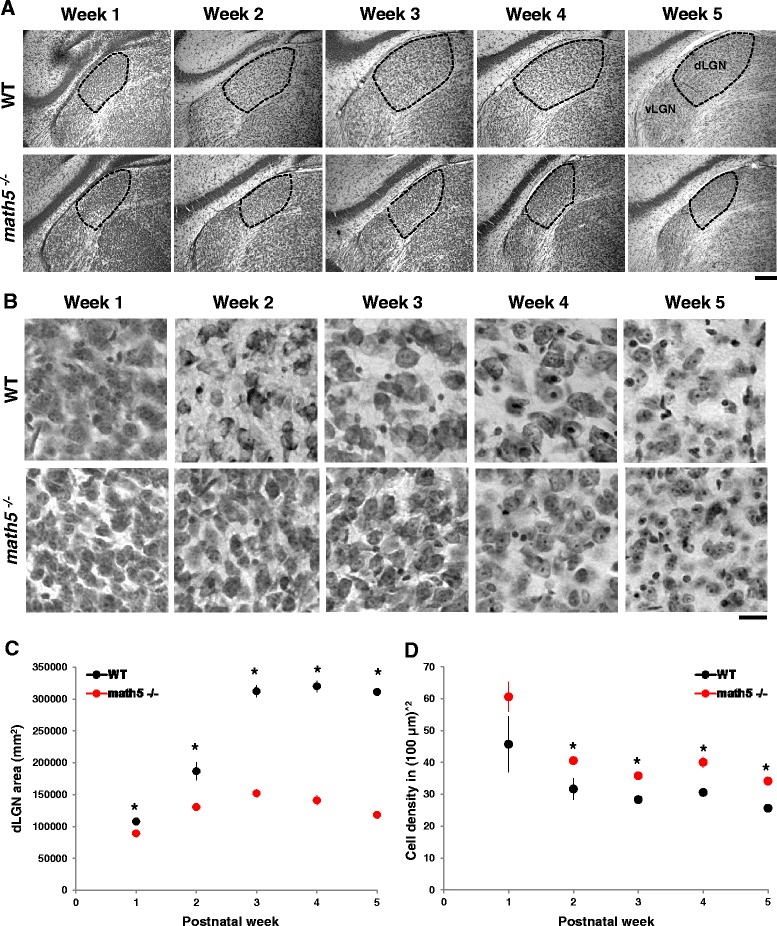
Cytoarchitecture of dLGN in WT and math5^−/−^. **a** Coronal sections of dLGN stained for Nissl at different postnatal weeks in WT (top panels) and *math5* ^*−/−*^ (bottom panels). In *math5* ^*−/−*^, dLGN boundaries are well delineated, but the nucleus is smaller compared to WT (vLGN: ventral lateral geniculate nucleus). Scale bar = 200 μm. **b** High power images of Nissl stained dLGN cells at different postnatal weeks in WT (top panels) and *math5* ^*−/−*^ (bottom panels). Scale bar = 20 μm. **c** Scatter plot depicting the mean dLGN area ± SEM as a function of postnatal week in WT (black) and *math5* ^*−/−*^ (red). Compared to WT, the dLGN of *math5* ^*−/−*^ was smaller at all weeks (*, weeks 1, 3–5 *p* <0.0001; week2 *p* <0.01). **d** Scatter plot showing cell density (±SEM) as a function of postnatal week in WT (black) and *math5* ^*−/−*^ (red). Compared to WT, cell density was significantly higher in *math5* ^*−/−*^ in weeks 2–5 (*, Student’s *t*-test, *p* <0.003)

First we examined whether the absence of retinal input in *math5*^*−/−*^ mice affected the overall growth and cytoarchitecture of the developing dLGN. We used Nissl stain to delineate the boundaries of dLGN from surrounding nuclei, and to measure the area of the dLGN. Figure 4a, shows examples of coronal sections at each week for WT (*n* = 51) and *math5*^*−/−*^ (*n* = 79). In WT mice, there was a 3-fold increase in dLGN area between postnatal weeks 1–3 (Fig. 4a,c; *n* = 33 week1, 10.8×10^2^ mm^2^ vs. *n =* 7 week3, 31.2×10^2^ mm^2^; Tamhane *post hoc* test, *p* <0.0001). Between three and five weeks the dLGN area remained stable (week 5, *n* = 4, 31.1×10^2^ mm^2^). In *math5*^*−/−*^, dLGN and surrounding nuclei were readily apparent (Fig. 4a, bottom row) and showed a 1.7-fold increase in area that peaked by postnatal week 3 (*n* = 35 week1, 8.9×10^2^ mm^2^ vs. *n* = 8 week3, 15.2×10^2^ mm^2^; Bonferroni *post hoc* test, *p* <0.0001). Between weeks 3–5 the size of dLGN decreased, so that by the fifth week, the area was comparable to postnatal week 1 values (*n* = 7, Bonferroni *post hoc* test, *p* <0.01). However compared to WT, *math5*^*−/−*^ dLGN was significantly smaller and after week 1 showed roughly a 50 % reduction in size (Student’s *t*-test; weeks1, 3–5 *p* <0.0001; week2 *p* <0.01).

We also used these Nissl stained sections to obtain estimates of cell density (Fig. 4b). In WT (*n* = 22 dLGN sections), there was no significant difference in the density of dLGN cells with age (Fig. 4b,d; Cell density *n* = 5 week1, 45.7 ± 8.8 cells/10^4^ μm^2^ vs. *n* = 3 week2, 31.7 ± 3.3 cells/10^4^ μm^2^; *n* = 6 week3, 28.3 ± 1.2 cells/10^4^ μm^2^; *n* = 4 week4, 30.6 ± 1.3 cells/10^4^ μm^2^; *n* = 4 week5, 25.6 ± 0.7 cells/10^4^ μm^2^; Student’s *t*-test, *p* >0.06). In *math5*^*−/−*^ (*n* = 38 dLGN sections), there was roughly a 40 % reduction in cell density between postnatal weeks 1–3 (*n* = 4 week1, 60.6 ± 4.7 cells/10^4^ μm^2^ vs. *n* = 7 week3, 35.8 ± 0.4 cells/10^4^ μm^2^; Student’s *t*-test, *p* <0.0001). Between weeks 3–5, cell density stabilized (*n* = 8 week5, 34.1 ± 1.3 cells/10^4^ μm^2^; Student’s *t*-test, *p* = 0.26). Compared to WT, cell density was significantly higher in *math5*^*−/−*^ at weeks 2–5 (*t*-test, *p* <0.003; week 1 Student’s *t*-test, *p* = 0.2). Thus for *math5*^*−/−*^, the reduction in dLGN size does not appear to be a consequence of cell loss.

### Morphological characteristics of developing relay cells in WT and math5^−/−^ mice

**Fig. 5:**
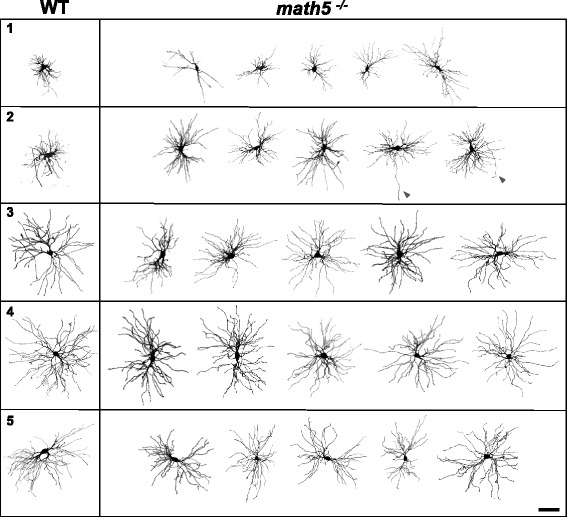
Biocytin filled relay cells in dLGN of WT and *math5* ^*−/−*^ *.* Examples of relay cells arranged by postnatal week (1–5) for WT (left panel) and *math5* ^*−/−*^ (right). Three-dimensional reconstructions are based on Z-stack images of representative relay neurons filled with biocytin. Arrowhead points to the axon. Scale bar = 58 μm

In order to examine whether the absence of retinal input influences the morphological development of relay cells we made in vitro recordings from acutely prepared slices containing the dLGN and filled cells with biocytin [30]. We then conducted multi-photon laser scanning microcopy to generate 3D reconstructions. Figure 5 shows representative examples of biocytin filled relay cells at different postnatal ages in WT and *math5*^*−/−*^ mice. At all ages examined, *math5*^*−/−*^ cells had large somata, multipolar dendrites, and axons that exit the nucleus (Fig. 5, arrowheads). Qualitatively, *math5*^*−/−*^ relay cells appeared similar to age matched WTs. However, quantitative analysis revealed a number of differences in their growth patterns and dendritic architecture.

### Somatic and dendritic surface area of relay cells

**Fig. 6:**
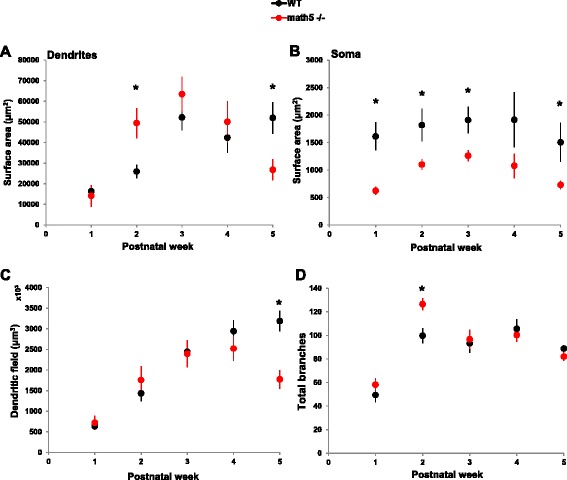
Morphological development of relay cells in WT and *math5* ^*−/−*^. Summary scatter plots depicting dendritic surface area (**a**), soma surface area (**b**), dendritic field (**c**) and total number of branches (**d**) for relay cells in WT (black) and *math5* ^*−/−*^ (red). Each point represents mean values ± SEM plotted as a function of postnatal age. **a** Dendritic surface area is greater in *math5* ^*−/−*^ than WT at week 2 (*, *p* <0.01), but smaller at week 5 (*, *p* <0.05). **b** Overall, soma surface area in *math5* ^*−/−*^ is less than WT (*, *p* <0.05). **c** WT and *math5* ^*−/−*^ have similar field dimensions during weeks 1–4, but *math5* ^*−/−*^ are smaller than WT at week 5 (*, *p* <0.01). **d**. At week 2, *math5* ^*−/−*^ cells exhibit an exuberant branching compared to WT (*, *p* <0.01)

Using the Volocity software, surface area was measured by highlighting the soma and dendrites in the X, Y and Z planes. In WT relay cells, there was a significant increase in dendritic surface area with age (Fig. 6a; *n* = 69 cells; one-way ANOVA, F = 9.103, *p* <0.0001). There was about a 3-fold increase between the first and third postnatal weeks (*n* = 8 week 1, 1.64×10^4^ ± 6.58×10^3^ μm^2^*vs. n* = 19 week3, 5.21×10^4^ ± 6.21×10^3^ μm^2^; Bonferroni *post hoc* test, *p* <0.0001). After this time, dendritic surface area showed no significant changes through postnatal week 5 (*n* = 8, 5.12×10^4^ ± 6.58×10^3^ μm^2^; Bonferroni *post hoc* test, p = 1). While dendritic area increased with age, soma surface area remained relatively constant throughout postnatal weeks 1–5. (Fig. 6b; one-way ANOVA, F = 0 .444, p = 0.777; see also [30].

In *math5*^*−/−*^ mutants (*n* = 55 cells), there was a steady increase in dendritic surface area that peaked by the third postnatal week and represented roughly a 4-fold increase compared to postnatal week 1 (Fig. 6a; *n* = 7 week1, 1.41×10^4^ ± 1.08×10^4^ μm^2^*vs. n* = 17 week3, 6.34×10^4^ ± 6.93×10^3^ μm^2^; Bonferroni *post hoc* test, *p* <0.01). However, between weeks 3 and 5 dendritic area declined, and was comparable to week 1 values (*n* = 8, 2.68×10^4^ ± 1.28×10^4^ μm^2^; Bonferroni *post hoc* test, *p* = 1). Soma surface area showed a similar growth pattern, increasing steadily through the third postnatal week but then showing a reduction in weeks 4–5 (Fig. 6b; *n* = 17 week3, 1.26×10^3^ ± 1.49×10^2^ μm^2^*vs. n* = 5 week5, 7.31×10^2^ ± 2.76×10^2^ μm^2^; Tamhane *post hoc* test, *p* <0.01).

Comparisons between WT and *math5*^*−/−*^ relay cells revealed that dendritic surface area was comparable during postnatal weeks 1, 3 and 4. However, *math5*^*−/−*^ relay cells showed a significant increase in dendritic surface area during postnatal week 2 (Fig. 6a; *n* = 25 WT, 2.59×10^4^ ± 3.73×10^3^ μm^2^ vs. *n* = 17 *math5*^*−/−*^, 4.94×10^4^ ± 6.93×10^3^ μm^2^; Student’s *t*-test, *p* <0.01). Such growth was not sustained and by the fifth postnatal week, relay cells in *math5*^*−/−*^ were significantly smaller than those in WT (Fig. 6a; *n* = 8 WT, 5.12×10^4^ ± 6.58×10^3^ μm^2^ vs. *n* = 5 *math5*^*−/−*^, 2.68×10^4^ ± 1.28×10^4^ μm^2^; Student’s *t*-test, *p* <0.05). Overall, soma surface area in *math5*^*−/−*^ mice was significantly smaller than WT (Fig. 6b, Student’s *t*-test, *p* <0.05; but see week 4 *n* = 9 WT, 1.91×10^3^ ± 3.87×10^2^ μm^2^ vs. *n* = 8 *math5*^*−/−*^, 1.08×10^3^ ± 2.18×10^2^ μm^2^; Student’s *t*-test, *p* = 0.9).

Relay cells in *math5*^*−/−*^ mice showed fluctuations in dendritic growth compared to WT, initially experiencing exuberant growth (week 1–3), followed by a progressive decline (week 4–5). To address whether these changes were due to the lengthening and sprouting of new branches (exuberant growth), or the shrinkage and pruning of dendrites (decline), we examined overall dendritic field, the number of branches, and branching patterns.

### Dendritic field

Using Volocity software, we measured the maximal dendritic extent in the X, Y, and Z axes, then multiplied the values to obtain an estimate of dendritic field. In WT, relay cells underwent a progressive increase in dendritic field size until the third postnatal week (Fig. 6c; *n* = 82; one-way ANOVA, F = 16.97, *p* <0.0001). Between weeks 3–5, fields stabilized, and overall showed a 5-fold increase compared to week 1 (Fig. 6c; *n* = 10 week 1, 6.35×10^5^ ± 2.93×10^5^ μm^3^*vs. n* = 10 week 5, 3.19×10^6^ ± 2.93×10^5^ μm^3^; Bonferroni *post hoc* test, *p* <0.0001).

In *math5*^*−/−*^ relay cells (*n* = 58), dendritic fields increased over the first 4 postnatal weeks and experienced a 3-fold increase compared to week 1 (Fig. 6c; *n* = 8 postnatal week 1, 7.20×10^5^ ± 4.18×10^5^ μm^3^ vs. *n* = 10 postnatal week 4, 2.52×10^6^ ± 3.74×10^5^ μm^3^; Tamhane *post hoc* test. *p* <0.01). By week 5, dendritic field values declined further but were not significantly different from week 4 (*n* = 5 postnatal week 5, 1.77×10^6^ ± 5.29×10^5^ μm^3^; Tamhane *post hoc* test, *p* = 0.12).

Compared to WT, *math5*^*−/−*^ relay cells showed comparable changes in dendritic field area throughout the first 4 postnatal weeks. However, *math5*^*−/−*^ relay cells were significantly smaller than WT at postnatal week 5 (Student’s *t*-test, *p* <0.01) suggesting arrested growth.

### Dendritic complexity

We examined the total number of dendritic branches and the pattern of branching for individual relay cells in the Z-plane by identifying primary dendrites, and their successive daughter branches [30].

In WT relay cells, the total number of branches increased with age (Fig. 6d; *n* = 40, one-way ANOVA, F = 9.67, *p* <0.0001). Between weeks 1 and 2, relay cells displayed a 2-fold increase in dendritic branches but then stabilized through weeks 2–5 (see also [30]). On average, WT cells had 49.4 ± 7.3 total branches during the first week and 98.2 ± 3.8 thereafter. In *math5*^*−/−*^ relay cells, dendritic branching also showed significant changes with age (Fig. 6d; *n* = 51, one-way ANOVA, F = 13.557, *p* <0.0001). Between weeks 1–2, branch numbers increased from a mean of 58.1 ± 7.7 (*n* = 8) to 126.5 ± 5.6 (*n* = 15; Bonferroni *post hoc* test, *p* <0.0001). However, this increase was transient, so that by the fifth week the number of dendritic branches was reduced to a mean of 82.0 ± 3.7 (*n* = 4; Bonferroni *post hoc* test, *p* <0.01). Moreover, when compared to WT, *math5*^*−/−*^ relay cells showed significantly higher numbers of dendritic branches during postnatal week 2 (Fig. 6d; *n* = 9 WT, 99.7 ± 6.9 vs. *n* = 15 *math5*^*−/−*^, 126.5 ± 5.6; Student’s *t*-test, *p* <0.01).

**Fig. 7:**
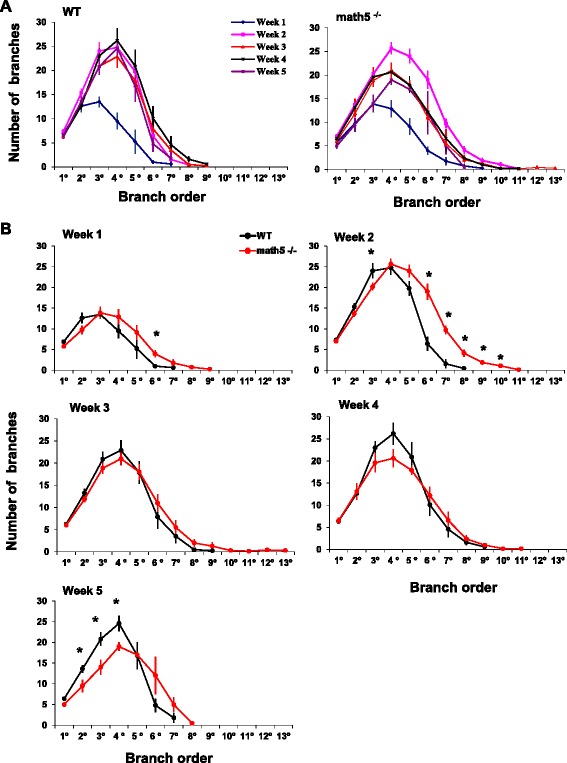
Analysis of dendritic branching patterns of relay cells for WT and *math5* ^*−/−*^. **a** Summary plots depicting the mean ± SEM number of branch points for relay cells as a function of branch order at different postnatal weeks (1–5) in WT (left panel) and *math5* ^*−/−*^ (right panel). In WT, branching patterns is conserved between weeks 2–5, but highly variable in *math5* ^*−/−*^ *.* **b** Comparison of the branching patterns between WT (black) and *math5* ^*−/−*^ (red) at different postnatal weeks. At week 1–2, *math5* ^*−/−*^ has increased numbers of higher order branching (6^th^-10^th^ order). At weeks 3–4, branching patterns are similar in WT and *math5* ^*−/−*^. At week 5, *math5* ^*−/−*^ has reduced numbers of 2^nd^-4^th^ order branches. (*, week 1, *p* <0.01; week 2, branch orders 6–8, *p* <0.0001 and branch orders 9–10, *p* <0.01; week 5*,* branch orders 2–4, *p* <0.05)

To examine dendritic branching patterns we calculated the number of branch points as a function of branch order. Figure 7a depicts summary plots for these relationships. At all ages both WT and *math5*^*−/−*^ cells had 6–7 primary dendrites, with the highest number of branching occurring between the 3^rd^-5^th^ orders. Week by week comparisons of branch complexity between WT and *math5*^*−/−*^ relay cells are shown in Fig. 7b. During week 1, *math5*^*−/−*^ relay cells showed increased numbers of 6^th^ order branches (*n* = 8 WT, 1.0 ± 0.4 vs. *n* = 8 *math5*^*−/−*^, 4.0 ± 0.9; Student’s *t*-test, *p* <0.01). Branch order continued to expand during week 2, so that *math5*^*−/−*^ cells had significantly more 6^th^ -10^th^ order dendritic segments compared to WT(Student’s *t*-test, branch orders 6–8 *p* <0.0001, branch orders 9–10 *p* <0.01). However, increased sprouting was transient, so that by weeks 3–4 there were no differences in the total numbers of dendritic branches or branch order compared to WT cells (Figs. 6d and 7b). Moreover, by week, 5, additional losses were observed among 2-4^th^ order segments (Fig. 7b; *n* = 5 WT vs. *n* = 4 *math5*^*−/−*^, Student’s *t*-test, branch orders 2–4 *p* <0.05).

In sum, these analyses show that the increased dendritic surface area noted in week 2 for *math5*^*−/−*^ relay cells is due to exuberant dendritic branching, especially among higher order segments (Figs. 6a, d and 7b). Furthermore, the reduction in dendritic surface area at week 5 is likely a consequence of attenuation in dendritic field as well as a continued loss of dendritic branches (Fig. 6a, c and 7b).

### Relay cell class specificity and location in math5^−/−^ dLGN

**Fig. 8:**
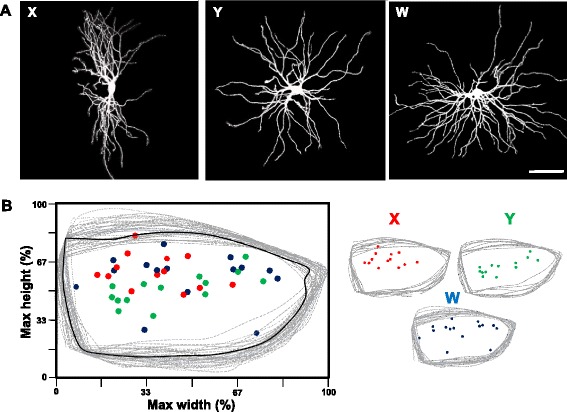
Relay cell class specificity is preserved in dLGN of *math5* ^*−/−*^. **a** Projection images of 3D rendered relay neurons in *math5* ^*−/−*^ showing the three morphologically identified classes of relay cells; *left*: X-like with bi-conical dendritic morphology; *middle:* Y-like with radially oriented dendritic arbors; and *right* W-like with hemispherical morphology. Classification is based on a DO*i* as described in [30] (Krahe et al., 2011). Scale bar = 58 μm. **b** Scatter plot depicting the location of relay cells in the dLGN of *math5* ^*−/−*^. Dashed lines represent superimposed coronal slices (300 μm thick) delineating the outlines of dLGN. Colors depict identified cell types (X-like: red; Y-like: green; W-like: blue)

Recently we showed that relay cells can be divided into three classes that have distinct dendritic architecture and strong regional preferences in dLGN [30]. This classification scheme was based on a Scholl ring analysis and the computation of a dendritic orientation index (DO*i*) that was based on the number of intersections found in each of four axial planes [30]. Cells with a DO*i* between 0–0.49 had a bi-conical morphology (X-like); those with values between 0.50-0.79 had a hemispheric profile (W-like), while those between 0.80-1.0 were radially symmetric (Y-like). Using the identical approach, we analyzed the dendritic architecture of 42 relay cells in *math5*^*−/−*^ dLGN. Similar to our previous study, we limited our analysis to postnatal weeks 2–5, at times when total dendritic branching stabilizes (Fig. 6d; see also [30]). Despite the transient increase in branching in week 2 and the subsequent loss in week 5, *math5*^*−/−*^ relay cells were of sufficient complexity to retain their identity. Figure 8a depicts representative examples (see also Fig. 5, weeks 2–5). A total of 13 cells were classified as Y-like with DO*i* values ranging between 0.81-0.97, 13 as X-like (DO*i* = 0.09-0.4) and 16 as W-like (DO*i* = 0.47-0.72).

Figure 8b depicts the relative position of identified X-, Y-, and W- like cells within the boundaries of the *math5*^*−/−*^ dLGN. Because of the reduced size of the *math5*^*−/−*^ dLGN, it became difficult to sample specific regions in an unbiased way during the recording, especially along the ventromedial border. Additionally, the associated compression makes it difficult to compare regional preferences with their age-matched WT counterparts. Nonetheless, similar to the regional preferences of cell types noted in WT dLGN [30], a qualitative examination of cell location in the *math5*^*−/−*^ dLGN revealed that Y-like cells resided in a central band throughout the nucleus and W-like cells were preferentially located along the dorsal border of the dLGN (Fig. 8b).

Finally, it is important to note that the changes in *math5*^−/−^ dendritic architecture noted at postnatal week two (Fig. 6) were unlikely restricted to a specific cell class. For *math5*^−/−^, each cell class showed an increase in branch number and dendritic surface area compared to WT counterparts (branch number *math5*^−/−^ vs WT: X-cells: *n* = 4 mean = 115.5 vs *n* = 2 mean = 88.5, Y-cells *n* = 3 mean = 121.0 vs *n* = 3 mean = 112.0, W-cells *n* = 8 mean = 134.2 vs *n* = 3 mean = 96.0; dendritic surface area *math5*^−/−^ vs WT: X-cells *n* = 5 mean = 37,036 vs *n* = 8 mean = 17,737, Y-cells *n* = 3 46,647 vs *n* = 11 mean = 25,227, W-cells *n* = 9 mean 54,807 vs *n* = 6 mean = 30,527) and had values that were similar to their respective group mean (Fig. 6a and d)

### Membrane properties of relay cells in math5^−/−^ dLGN

**Fig. 9:**
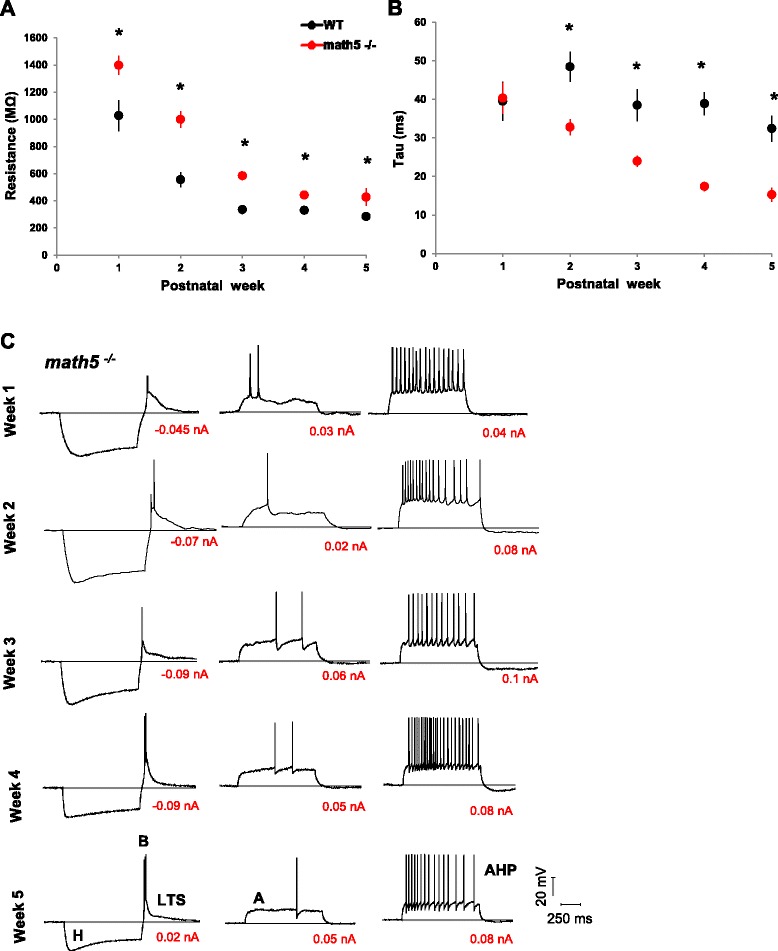
Passive and active membrane properties of relay cells in WT and *math5* ^*−/−*^. **a** Scatter plot showing mean input resistance ± SEM in WT (black) and *math5* ^*−/−*^ (red) at different postnatal weeks. Estimates based on steady state voltage responses evoked by −0.01 nA current pulse. Overall, *math5* ^*−/−*^ have higher input resistance than WT (*, week 1, 4–5 *p* <0.05, week 2–3 *p* <0.0001). **b** The decay constant tau as determined by a single exponential fit to a −0.01 nA current injection for the first 200 ms of recording. *Math5* ^*−/−*^ (red) have a shorter τ than WT (black, *, *p* <0.0001). **c** Examples of the voltage responses to current injection for *math5* ^*−/−*^ relay cells at different postnatal weeks. *Math5* ^*−/−*^ relay cells possess the full complement of membrane properties as in WT. These include a depolarizing sag mediated by a mixed cation conductance (**H**), a rebound low threshold Ca^2+^ spike (**LTS**) and burst firing (**B**), an outward rectifying response that delays spike firing (**A**), and spike frequency accommodation (**AHP**)

The intrinsic membrane properties of relay cells in the *math5*^*−/−*^ dLGN were examined by conducting *in vitro* whole cell recordings prior to filling them with biocytin. We recorded the voltage responses to square wave current pulses of varying duration and intensity delivered through the recording pipette (e.g. ± 0.01 nA, 1000 ms, 0.0025 nA increments). The passive properties of input resistance and tau (τ) were calculated by examining the voltage response to a small hyperpolarizing current pulse (−0.01 nA). As in WT (*n* = 116), *math5*^*−/−*^ relay cells (*n* = 133) showed a decrease in input resistance with age (Fig. 9a). However, input resistance was significantly higher in *math5*^*−/−*^ cells at all studied ages compared to WT (Fig. 9a; Student’s *t*-test, week 1, 4–5 *p* <0.05, week 2–3 *p* <0.0001). These differences can be in part explained by the reduced soma area seen in *math5*^*−/−*^ (Fig. 6b). In WT cells (*n* = 51), τ remained relatively stable with age (Fig. 9b), but showed a progressive decrease in *math5*^*−/−*^ (*n* = 61). Compared to WT, *math5*^*−/−*^ had similar τ during week 1 (*n* = 8 WT, 39.5 ± 5.1 ms vs. *n* = 8 *math5*^*−/−*^, 40.3 ± 4.02 ms; Student’s *t*-test, *p* = 0.09), but then became significantly shorter between weeks 2–5 (Student’s *t*-test, week 2–5 *p* <0.01). Such a pattern may be due to the smaller dendritic fields observed in *math5*^*−/−*^ (Fig. 6a and c).

Examples of voltage responses to current steps in *math5*^*−/−*^ cells are shown in Fig. 9c. Many of the voltage-gated conductances noted in WT were also present in *math5*^*−/−*^ age matched cells (not shown but see [19, 28, 30, 36]). For example in *math5*^*−/−*^ relay cells, membrane hyperpolarization evoked a strong inward rectification. This large depolarizing sag in the voltage response reflects the activation of the mixed cation conductance (H) [28, 32]. In addition, the termination of membrane hyperpolarization activated a t-type Ca^2+^ conductance that produced a rebound low-threshold calcium spike (LTS), along with a burst of Na^+^ spikes that ride the peak of this triangular depolarization. With membrane depolarization, relay cells exhibited an outward rectification that delayed spike firing and reflected the activation of a transient (A) type K^+^ conductance [32, 36, 38]. Strong and sustained levels of membrane depolarization readily evoked a train of action potentials that exhibited spike frequency accommodation, an event attributed to the activation of K^+^ conductances that produce an after hyperpolarizing response between spikes (AHP) [32, 36].

Overall, these observations indicate that the intrinsic membrane properties and spike firing of relay cells remain largely unaffected by the absence of retinal innervation.

## Discussion

**Fig. 10:**
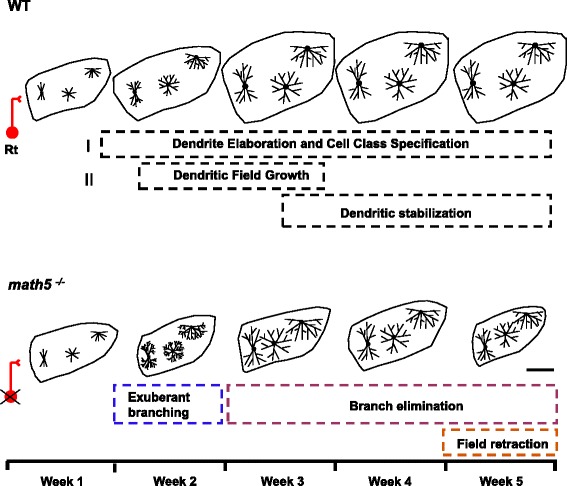
Development of relay cells in WT and *math5* ^*−/−*^. Schematic summarizing the development of relay cells in WT (top row) and *math5* ^*−/−*^ (bottom row). Roman numerals (I, II) represent identified growth spurts in WT. Highlighted boxes depict the nature and timing of growth. Outlines of dLGN are drawn to scale, 200 μm

Our data from WT mice suggest that dLGN relay cells undergo two growth spurts [43]. The major elements and timing of these events are outlined in Fig. 10. The first phase takes place during postnatal week 1, as dendritic branches increase in number and grow in length to form highly stereotypic architecture and cell class specificity [30]. The second phase occurs during postnatal weeks 2–3 where there is a progressive increase in dendritic field size. During this time no additional branch elaboration occurs and the overall complexity of dendritic arbors remains stable. The timing of these growth spurts corresponds to a progressive increase in dLGN size, and like the maturation of relay cells, the nucleus assumes an adult-like profile by postnatal week 3 [25].

Our results in *math5*^*−/−*^ mutants indicate that the absence of retinal innervation disrupts the normal growth and maturation of dLGN relay cells (Fig. 10). Initially relay cells in *math5*^*−/−*^ follow a similar growth trajectory as age matched WT cells. However, they undergo an extended period of branch elaboration, showing an increase in branch number and length throughout postnatal week 2. Such exuberant branching is not maintained. In fact, the total number of branches and overall branch order declines through postnatal week 5, and leads to an overall reduction in dendritic surface area. Accompanying these dystrophic changes is shrinkage in the overall size of dLGN.

Taken together, these data suggest that retinal innervation plays an important trophic role in dLGN development. Indeed, the development of the retinogeniculate pathway seems to satisfy many of the key elements for “synaptotrophic” support (reviewed in [56] and [14]). A major tenant for such support is that dendritic development and synapse formation/maturation are concurrent. Retinal axons arrive in dLGN at perinatal ages, and by early postnatal life fully innervate the dLGN [22, 28, 42]. Soon after innervation retinal axons form functional synapses with developing dLGN cells [5, 28]. These newly formed synapses are of sufficient excitatory strength to drive action potentials in dLGN relay cells [5, 28, 33, 40]. Such excitatory drive is provided by spontaneous wave like activity of RGCs that prevail prior to the onset vision [18, 35, 40]. Additionally, dendritic maturation of relay cells coincides with a highly active period of synaptic remodeling and maturation. Structurally, retinal profiles (RLP) expand in size and complexity, showing a dense clustering of vesicles [5]. Functionally, developing dLGN cells receive relatively weak synaptic input from as many a dozen or so RGCs [13, 28, 64]. By postnatal week 2 many of these inputs are eliminated and the remaining few show a substantial increase in synaptic strength, as well as a shift in NMDA to AMPA receptor composition (reviewed in [23, 26, 33]).

Our results in *math5*^*−/−*^ mutants also suggest that retinal innervation is needed for constraining and stabilizing the dendritic complexity of relay cells. It is believed that developing dendrites sample their environment and extend processes into regions where prospective synaptic afferents are found (reviewed in [14, 15, 57]). Perhaps the extensive dendritic branching we observed in *math5*^*−/−*^ relay cells reflects a compensatory response by these cells to seek potential synaptic partners. This notion is consistent with other reports showing that developing neurons alter their dendritic form in response to a disruption in afferent input (reviewed in [12, 37]). Finally it is worth noting that retinal signaling is required for the continued maintenance of dendritic form. In *math5*^*−/−*^ relay cells, the exuberant branching observed during postnatal week 2 is eliminated and followed by a modest decline in proximal dendritic segments.

Our results suggest that RGCs provide trophic support that sustains the development of relay cells, as well as to support the overall structural integrity of dLGN. Such trophic effect for retinal axons on the growth of dLGN has been previously described in enucleation and anophthalmic studies where distortion and shrinkage of dLGN have been reported [16, 25, 62]. However, the molecular mechanisms underlying trophic support and maintenance of the mouse dLGN remain largely unknown. A likely candidate is the brain-derived neurotrophic factor (BDNF). This neurotrophin is synthesized in the retina, transported anterogradely by retinal afferents and can bind to their high affinity receptor tyrosine kinase (trkB) located on dLGN dendrites [2, 11, 15, 39].

Finally it is important to note that despite the disruption in growth and maturation, relay cells in the *math5*^*−/−*^ dLGN still retained a high degree of branch complexity, morphological class specificity, and the full complement of active membrane properties. Such observations suggest that dendritic form and function are likely regulated by other factors unrelated to retinal innervation and signaling. One possibility is that synaptic signaling from non-retinal inputs could provide additional trophic support. Indeed the bulk of synaptic input to dLGN arise from a number of non-retinal sources, including glutamatergic neurons in layer VI of visual cortex, cholinergic nuclei of the brainstem, and GABAergic neurons within the thalamic reticular nucleus as well as intrinsic interneurons within the dLGN [5, 46, 49]. Many of these elements have been implicated in supporting developing dendritic form and function [3, 17, 34, 50, 59] (reviewed in [4]). Most notable are the inputs that arise from visual cortex, where the infusion of neurotrophic factors leads to an accelerated growth of relay cells [59]. Interestingly in mouse, corticogeniculate inputs arrive at late postnatal ages, well after retinal innervation [46]. Such timing suggests that these descending projections are poised to contribute to the maintenance and stability of dendritic form. In fact, in the *math5*^*−/−*^*,* dLGN cortical inputs arrive much earlier than in WT [7, 46], and thus could help explain why relay cells in these mutants retain much of their overall structural and functional integrity.

## Conclusions

The dLGN of mouse has proven to be an important model system for visual circuit development. However there is a paucity of information regarding the development of its principal cell type, namely thalamocortical relay cells. Here we examined the postnatal growth and maturation of dLGN relay cells and tested, by utilizing *math5*^*−/−*^ mice, the extent to which their dendritic form and function relied on retinal innervation. We found that the absence of retinal innervation leads to an overall shrinkage of dLGN and disrupts the pattern of dendritic growth of relay cells. In *math5*^*−/−*^ dLGN, relay cells undergo a period of exuberant dendritic growth and branching followed by branch elimination and an overall attenuation in dendritic field size. Despite these dystrophic changes, relay cells in *math5*^*−/−*^ mice retained a sufficient degree of complexity and cell class specificity, as well as the full complement of membrane properties and spike firing characteristics. Thus retinal innervation plays an important trophic role in dLGN development, but that additional support perhaps arising from non-retinal innervation and signaling, contributes to stabilization of dendritic form and function.

## Methods

### Subjects

All procedures carried out were approved by the Institutional Animal Care and Use Committee at Virginia Commonwealth University. Mice ranging in age between the first and fourth postnatal weeks were studied. Two strains were used: pigmented wild-type mice (C57/BL6), *math5*^*−/−*^ on a mixed C57B6/J and 129/SvEv background provided by S. Wang [60].

### CTB injection

Injection of the anterograde tracer cholera toxin subunit beta (CTB) were performed in order to visualize retinal projections in the dLGN and to assess whether any surviving RGCs in *math5*^*−/−*^ had axons that exited the eye and innervated retino-recipients targets in the brain. Mice were anesthetized with isoflurane vapors. Using a glass pipette, the sclera was pierced near the ora serrata and excess vitreous fluid was drained. Using another glass pipette attached to a picospritzer, 3–8 μl of CTB (1.0 % solution dissolved in distilled water) conjugated to Alexa Fluor 488 or 594 dyes (Invitrogen) were then injected into the same opening used to drain the excess vitreous fluid. Following eye injections, animals were given a 2-day survival period to allow the tracer to travel to central visual targets such as SCN or dLGN.

### Acute in vitro thalamic slice preparation

Whole cell recording and filling of relay cells were done using methods described elsewhere [5, 19, 28, 30]. Animals were anesthetized with isoflurane and decapitated. The brain was excised and placed in a 4 °C oxygenated (95 % O_2_/5 % CO_2_) slicing sucrose solution (in mM: 26 NaHCO_3_, 23.4 sucrose, 10 MgSO_4_, 0.11 glucose, 2.75 KCl, 1.75 Na H_2_PO_4_, 0.5 CaCl_2_). Slices (300 μm) were cut in the coronal or parasagittal planes on a vibratome (Leica VT1000S), and placed for 1 h in a 35 °C oxygenated solution of artificial cerebral spinal fluid (ACSF) (in mM: 124 NaCl, 2.5 KCl, 1.25 NaH_2_PO_4_, 2.0 MgSO_4_, 26 NaHCO_3_, 10 glucose, 2 CaCl_2_). Slices containing dLGN were selected for *in vitro* intracellular recording in the whole cell current clamp mode, and were perfused in an oxygenated solution of ACSF that was kept heated at 30 °C. Cells were visualized with the aid of IR-DIC optics, and were patched with electrodes made of borosilicate glass filled with an internal solution (in mM: 140 K gluconate, 10 HEPES, 0.3 NaCl, 2 ATP-Mg, 0.1 GTP-Na; pH 7.25) containing 5 % biocytin. Patch electrodes were vertically pulled and had a final tip resistance of 3–7 MΩ. Electrodes were connected to an amplifier (Axoclamp 2B, Axon instruments). Different protocols of square wave current pulses were applied and the resulting voltage responses were measured. Neuronal activity was digitized with an interface unit (National Instruments) and stored on a computer. Data acquisition and analysis was done using Strathclyde Electrophysiology Software, Whole Cell Analysis Program V3.8.2.

At the end of the recording, slices were fixed overnight with 4 % paraformaldehyde (PFA) in 0.1 M phosphate buffer solution (pH = 7.2). To visualize dLGN cells filled with biocytin, slices were washed with phosphate buffer saline (PBS) (3×, 30 min), and incubated overnight at 4 °C in a PBS solution containing 0.1 % Triton X-100 and 0.1 % Alexa Fluor 647 conjugated streptavidin (Invitrogen). Slices were washed with PBS, mounted with ProLong Gold with DAPI (Invitrogen), and cured overnight at room temperature.

### Reconstruction of biocytin filled relay cells

Three-dimensional reconstructions and analysis were done using methods described previously [30]. Biocytin filled relay cells were imaged using a multi-photon laser-scanning microscope (Zeiss LSM510 NLO Meta). A HeNe laser (633 nm) was used to excite fluorescence from biocytin filled dLGN neurons and emission was detected at a range of 651–694 nm (Meta detector). The following objective lenses were used to image targeted neurons at a scanning resolution of 2048×2048 pixels: Plan-Neofluar 40× (1.3 n.a) oil immersion objective lens, or a C-Apochromat 40× (1.2 n.a) water immersion objective lens. 3-D datasets were compiled from a sequential series of optical slices with a step size through the Z-axis of 0.48 μm (40×/1.2 n.a. lens) or 0.5 μm (40×/1.3 n.a. lens). 3-D Z-stack datasets were analyzed using Volocity software (Improvision, version 4.3.2). Image sequences were deconvolved to reduce signal noise generated from outside the focal plane of interest using Iterative restoration technique, and thresholding values were set according to signal intensity and background noise.

### Cresyl violet nissl stain

Animals were anesthetized with isoflurane vapors, and transcardially perfused with PBS solution for 5 min, followed by 4 % paraformaldehyde in 0.1 M PBS (ph = 7.2) for 15–20 min. Brains were excised and fixed overnight with 4 % PFA. Slices containing dLGN were cut on a coronal plane with a vibratome (70 μm), and left to dry overnight. Slices were washed for 3 min in 95 % and 75 % ethanol solutions, respectively. Slices were washed in dH_2_O for 1 min, before immersing them in cresyl violet stain for 20–30 s, and then were rinsed briefly with dH_2_O. Sections were washed for 3 min in 70 %, 95 %, 95 %, 100 %, 100 % ethanol solutions, respectively. Finally, slices were washed in xylene twice for 5 min. Slides were mounted with Permount, and visualized with light microscopy (Olympus 1×71, Photometrics Cool snap camera), and pictures were taken with a 10× objective lens. Images were analyzed with Metamorph software. Area measurements were obtained from 2–4 consecutive sections through the middle of the dLGN. Nissl stained cell counts were calculated from a 100μm×100μm region of interest centered in the middle of 2–3 dLGN sections. Measurements were restricted to cells in which the soma and nucleus were clearly delineated.

### Enucleation

Binocular enucleation was done using methods described previously [46]. The eyes were removed after cutting the optic nerve and the ophthalmic artery. To avoid hemorrhaging, the orbit was filled with Gelfoam (Upjohn), and animals were allowed to recover on a heating pad.

### Immunohistochemistry

Slices containing dLGN were processed using antibody that stains for VGluT2, a vesicular glutamate transporter found in retinal terminals [61]. On a vibratome, 40 μm thick slices were cut on the coronal plane. Before incubation, sections were rinsed in PBS, and then treated for 1 h with blocking solution (5 % NGS, 2.5 % BSA and 0.1 % Triton X-100). Sections were incubated overnight with the primary antibody at 4 °C (rabbit anti-VGluT2: 1:1000, Synaptic Systems). Sections were rinsed with PBS, and were incubated in the secondary antibody (1:1000 dilution; Alexa 594 conjugated goat anti-rabbit IgG: 1:1000, Invitrogen, Cat# A11037) for 2 h at room temperature. Sections were rinsed in PBS, mounted with Prolong Gold with DAPI (Invitrogen) and cured overnight at room temperature. Sections were photographed with an upright epi-fluorescence microscope (Nikon E600, Photometrics Cool snap camera).

### RT-PCR

Retina and dLGN tissue were harvested from C57/BL6 mice at different embryonic and postnatal ages using methods described elsewhere [51]. RNA was isolated using the Bio-Rad Total RNA Extraction from Fibrous and Fatty Tissue kit (Bio-Rad). Reverse transcription and cDNA generation were made using Superscript II Reverse Transcriptase First- Strand cDNA Synthesis kit (Invitrogen). The following *math5* primer pairs were used: 5′- ATGGCGCTCAGCTACATCAT- 3′ and 5′-GGGTCTACCTGGAGCCTAGC- 3′.

### Electron microscopy

Ultrastructural analysis of dLGN was carried out as previously reported [5]. Mice (P21-22) were deeply anesthetized with isoflurane vapors and perfused transcardially with 2 % PFA/2 % glutaraldehyde in 0.1 M phosphate buffer solution. Brains were excised and cut on a coronal plane (50–100 μm thick) using a Vibratome (Leica VT100E). Sections were postfixed in 2 % osmium tetroxide, dehydrated in a graded series of ethyl alcohol and then were embedded in Durcupan resin. Ultrathin sections (70 nm) were cut, collected on Formvar-coated nickel slot grids and then were stained to reveal the presence of gamma amino butyric acid (GABA), using a polyclonal, affinity-purified rabbit anti-GABA primary antibody (cat. no. A2052, Sigma, St. Louis, MO) diluted 1:2,000, and a goat anti-rabbit IgG antibody conjugated to 15-nm colloidal gold particles diluted 1:25 (British BioCell International, Cardiff, UK). The sections were then stained with uranyl acetate and examined using a Philips CM10 electron microscope. Images of *math5*^*−/−*^ tissue (*n* = 20, P22) and WT tissue (*n* = 20, P21) were collected with a digitizing camera (SIA-7C; SIA, Duluth, GA). In each sample of images, all nonGABAergic terminal profiles were measured using the SIA software.

## Abbreviations

bHLH: Basic helix loop helix
BDNF: Brain-derived neurotrophic factor
CTB: Cholera toxin subunit B
DO*i*: Dendritic orientation index
dLGN: Dorsal lateral geniculate nucleus
E: Embryonic
GABA: Gamma-aminobutyric acid
*Gapdh*: Glyceraldehyde-3-phosphate dehydrogenase
*math5*^*−/−*^: *math5* null mutant
P: Postnatal
PBS: Phosphate buffer saline
PFA: Paraformaldehyde
RGC: Retinal ganglion cells
RLD: Round, large, dark mitochondria
RLP: Round, large, pale mitochondria
SCN: Suprachiasmatic nucleus
τ: tau
vLGN: Ventral lateral geniculate nucleus
VGluT2: Vesicular glutamate transporter 2
WT: Wild type
